# Virtual Reality Representations of Nature to Improve Well-Being amongst Older Adults: a Rapid Review

**DOI:** 10.1007/s41347-021-00195-6

**Published:** 2021-03-05

**Authors:** Josca Van Houwelingen-Snippe, Somaya Ben Allouch, Thomas J. L. Van Rompay

**Affiliations:** 1 University of Twente, Enschede, The Netherlands; 2 Amsterdam University of Applied Sciences, Amsterdam, The Netherlands

**Keywords:** People-environment interaction, Digital nature, Social well-being, Connectedness, Human-technology interaction

## Abstract

Poor well-being amongst older adults poses a serious health concern. Simultaneously, research shows that contact with nature can improve various facets of well-being, including physical, social, and mental well-being. However, nature is not always accessible for older adults due to mobility restrictions and related care needs that come with age. A promising strategy aims at bringing nature inside through pervasive technologies. However, so far, there is little academic understanding of essential nature characteristics, psychological processes involved, and means for implementation in practice. The current study used a three-folded rapid review to assess current understanding and strategies used for improving well-being for older adults through virtual reality representations of nature. Searches were performed across three databases, followed-up by content-based evaluation of abstracts. In total, a set of 25 relevant articles was identified. Only three studies specifically focus on digital nature as an intervention strategy for improving well-being amongst older adults. Although these studies provide useful starting points for the design and (technological) development of such environments, they do not generate understanding of how specific characteristics of virtual nature representations impact social well-being measures in particular, and of the underlying psychological processes involved. We conclude that follow-up research is warranted to close the gap between insights and findings from nature research, gerontology, health research, and human-technology interaction.

## Introduction

Poor well-being amongst elderly is increasingly recognized as a serious public health concern (Cacioppo et al., 2017; Hawkley & Cacioppo, 2007; Sorkin et al., 2002). Well-being comprises several domains, including physical, mental, social, and economic well-being (OECD, 2013). Research shows that interaction with nature can improve a wide range of well-being facets including happiness, positive affect, feelings of connectedness, and a sense of meaning in life (Bratman et al., 2019; Kaplan & Kaplan, 1989; Maas et al., 2009; van Houwelingen-Snippe et al., 2020a, b). However, nature is often not accessible for older adults because of mobility issues or because nature is becoming increasingly scarce in urbanized regions. Interestingly, recent developments underscore the potential of modern technologies including virtual and augmented reality for bringing nature inside. At the same time, research shows that indirect encounters with nature (such as watching videos of nature scenes) can already improve well-being (Baños et al., 2012). Taking note of advances in technology development and recent research findings from the social sciences, this review aims at identifying research studying the interplay between digital nature and well-being amongst older adults.

## Well-Being amongst Elderly

Poor well-being poses a serious public health concern (Cacioppo et al., 2017). Poor well-being may lead to mental health issues (i.e., depression, loneliness, and mood disorders) and physical health issues, including greater risk of cardiovascular disease (Sorkin et al., 2002) and accelerated physiological decline (Hawkley & Cacioppo, 2007). There is a long debate going on in the literature about the conceptual clarity of the concept of well-being. In this study, we define well-being as a complex, multilevel and multidimensional concept in which well-being is regarded as a state of equilibrium between elements within the body (e.g., bodily rhythms and processes) and external influences operating outside the body (e.g., social context, atmosphere, and the physical environment). Hence, well-being is a dynamic process that is affected by life events and (social) challenges (e.g., shrinking networks that come with old age) that humans continuously face (Dodge et al., 2012; Ng & Fisher, 2013; Fiorini et al., 2016; OECD, 2019a). In general, people experience high levels of well-being when they have the resources needed to meet and manage life’s challenges (Dodge, et al., 2012; Fiorini et al., 2016).

### Nature and Well-Being

A growing body of literature underscores the positive effects of nature experience on well-being, as also evidenced by several systematic reviews (Annerstedt & Währborg, 2011; Bratman et al., 2019; Gascon et al., 2015; Hunter et al., 2019). For instance, urban green space interventions can improve health and social benefits (Hunter et al., 2019) and being close to, or living in, nature can also reduce feelings of loneliness and boost perceptions of social support (Maas et al., 2009). Other reviews (Bratman et al., 2019; Gascon et al., 2015) point out that research is needed to identify causal links between nearby green space and (mental) well-being (i.e., what are underlying mechanisms and key properties of nature spaces that promote better mental health?), and to clarify the relationship between exposure duration and frequency of visits and effects obtained (Gascon et al., 2015). A cross-disciplinary body of evidence (including research from social and health sciences) stresses the importance of nature experience on mental well-being (Bratman et al., 2019). Based on this evidence, a conceptual model is presented to disseminate insights amongst stakeholders (such as city planners or architects) in order to raise awareness of the impact of urban planning decisions on mental well-being (Bratman et al., 2019).

In line with findings from these reviews, it has been shown that nature-based therapies (e.g., horticultural or wilderness therapy) can be effective and may complement therapy programmes for a variety of mental and physical diagnoses, such as dementia and obesity (Annerstedt & Währborg, 2011). Concluding, a considerable body of research documents the link between contact with nature (and related dimensions such as accessibility and availability of nature) and well-being. In the next section, studies focusing on elderly and nature interaction will be discussed.

### Elderly and Nature

Nature interaction seems to be beneficial for everyone. However, contact with nature and the close proximity of nature play a particularly important, yet nuanced, role in older adults’ everyday life (Finlay et al., 2015). By consequence, a relatively large body of research focuses on well-being benefits of nature for older adults (Detweiler et al., 2012; Kabisch et al., 2017; Wen et al., 2018).

In general, older adults benefit from green space as illustrated by a positive association between the availability of green space and perceived general health (Kabisch et al., 2017). According to a systematic review (including 44 articles) on the needs and preferences of older adults (Wen et al., 2018), older adults who engage in recreational activities in green spaces particularly value naturalness, aesthetics, and variety within the scene. Furthermore, for logistical reasons and safety considerations, accessibility of the green space and the inclusion of well-maintained paths are crucial for older adults to enjoy nature’s benefits (Wen et al., 2018). In line with the importance of being active in nature, therapeutic gardens and horticultural therapy have also been pointed out as particularly suited to older adults in general (Detweiler et al., 2012; Milligan et al., 2004), and to people living with dementia in particular (Hernandez, 2007; Murphy et al., 2010).

Various reviews have been undertaken focusing on social well-being, and loneliness in particular, amongst older adults (Landeiro et al., 2017) and possible interventions for reducing it (Dickens et al., 2011a; Fakoya et al., 2020). Factors predicting loneliness are widowhood, older age, poor mental or physical health, and being new in a community (De Koning et al., 2017). The experience of loneliness varies greatly across individuals, which makes it extremely challenging, if not impossible, to design a one size fits all loneliness intervention, according to a recent scoping review on 33 review articles (Fakoya et al., 2020). According to another systematic review (Dickens et al., 2011b), successful and effective interventions targeting social isolation share three characteristics: theory-informed (i.e., evidence-based) development, provision of social activity, and/or group support. Additionally, an active lifestyle also seems to increase effectiveness of interventions targeting social isolation in older adults (Dickens et al., 2011b). These research endeavours testify to the ongoing search for effective interventions promoting social well-being by decreasing loneliness and social isolation amongst older adults.

To sum up, we discussed a number of review articles focusing on the beneficial effects of nature on well-being (Annerstedt & Währborg, 2011; Bratman et al., 2019; Gascon et al., 2015), on social well-being amongst elderly (De Koning et al., 2017; Dickens et al., 2011a; Fakoya et al., 2020; Landeiro et al., 2017) and on the importance of nature interaction for promoting well-being of older adults in particular (Kabisch et al., 2017; Wen et al., 2018). In the present review, we aim to identify articles that focus on the cross sections of these topics: the effects of nature interaction on well-being for older adults.

### Virtual Reality Representations of Nature

Older adults do not always have access to nature and hence cannot enjoy nature’s benefits. When considering how to make nature accessible for people with limited or no access to nature, studies looking into the effects of virtual reality representations of nature are of particular interest. Research on the comparison of real-life nature interaction and virtual nature interaction indicates that simulated nature may exert similar benefits when compared with real nature (Annerstedt et al., 2013; Browning et al., 2020; Kjellgren & Buhrkall, 2010). Promising examples in health care research are augmented biking exercises with augmented nature (Bruun-Pedersen et al., 2014; Bruun-Pedersen et al., 2016; Grani & Bruun-Pedersen, 2017) or virtual nature in nursing homes for recreational purposes (Bruun-Pedersen et al., 2015a; Ludden et al., 2019). In short, these combined findings underscore the potential of virtual nature for enhancing diverse facets of well-being.

In the review study described next, we performed three rapid reviews to identify existing studies investigating the effects of digital nature on well-being for elderly. There has been considerable attention for the individual topics under investigation, and even for cross-topic combinations (e.g., assistive technology to reduce loneliness amongst older adults [Jansen-Kosterink et al., 2018; Ring et al., 2013; Ten Bruggencate et al., 2018; Zamir et al., 2018]). However, in this review, we are specifically interested in multi-disciplinary research aimed at integrating findings from nature studies, human technology interaction, and social and health-related studies. Therefore, the aim of the present review is to identify articles that focus on the effects of nature interaction on well-being for older adults. On top of that, the second aim of this review is to identify articles focussing on virtual reality representations of nature for older adults as a means to improve social well-being in particular. On the one hand, there are many creative technology applications and initiatives aimed at implementation of virtual reality representations of nature in various care settings. However, most of these are not evidence or theory-based. On the other hand, studies from the social sciences are revealing about the psychological processes involved. However, these studies usually do not aim at facilitating the bridge from science to practice. Combining these disciplines will open up new possibilities for health innovations.

## Methods

### Search Strategy

A rapid review was performed, to assess what is already known about Well-being, Elderly, Technology and Nature. A rapid review method is one of the review methods which fall under the umbrella of Cochrane Review Methods (Moher et al., 2015; Garritty et al., 2016). A rapid review has been described as evidence synthesis that uses methods to streamline those of systematic reviews to complete the evidence synthesis in a shorter turnaround time than a standardized systematic review (Gannan et al., 2010; Khangura et al., 2014; Polisena et al., 2015). Furthermore, a rapid review follows many of the principal steps of a systematic review, using systematic and transparent methods to identify, select, and critically analyse data from the relevant databases but the main difference is that some of the elements of a rapid review are either simplified or omitted, such as for example using one reviewer or reducing the number of used databases (HEARD, 2018). For this study, we used the online databases Scopus, Web of Science, and PubMed and did not include for example the IEEE database. Only studies written in the English language were considered.

#### Search Part 1

The search key words used in the study are presented in Table 1 arranged per topic.

**Table 1:** Search key words per topic Search Part 1

Elderly	Technology	Well-being	Nature
“Elderly”	Technolog*	“Wellbeing”	“Nature experience”
“Aged”	“System”	“Well-being”	“Nature exposure”
“Older”	“Virtual Reality”	“Positive mental wellbeing”	“Restorative nature”
“Elder”	“VR”	“Positive mental well-being”	“Nature environment”
“Geriatric”	“Augmented reality”	“Subjective wellbeing”	“Green space”
“Elderly people”	“Ambient technology”	“Subjective well-being”	“Blue space”
“Old people”	“Pervasive technology”	“Psychological wellbeing”	
“Senior”		“Psychological well-being”	
		“Emotional wellbeing”	
		“Emotional well-being”	
		“Social connectedness”	
		“Social isolation”	
		“Social wellbeing”	
		“Social well-being”	
		“Connectedness”	
		“Loneliness”	
		“Social isolation”	
		“Mental health”	
		Psycholog*	

For all databases, all combinations of the search key words were used. All synonyms per topic were connected with a disjunction (“elderly” OR “aged” OR “older” etc.) and all topics were connected with a conjunction (Elderly (and all synonyms) AND Technology (and all synonyms) etc.).

A content analysis was performed on those articles that were selected based on the combination of all topics (Elderly and Nature and Well-being and Technology). Two reviewers performed the search and reviewed the selected articles. Each reviewer decided whether (1) each abstract concerned the experience of [or interaction with] nature, (2) whether the study used digital representations of nature, and (3) whether the focus was on (a dimension of) well-being. Articles that described participant groups which included participants aged 65 and older were included.

#### Results Part 1

**Fig. 1:**
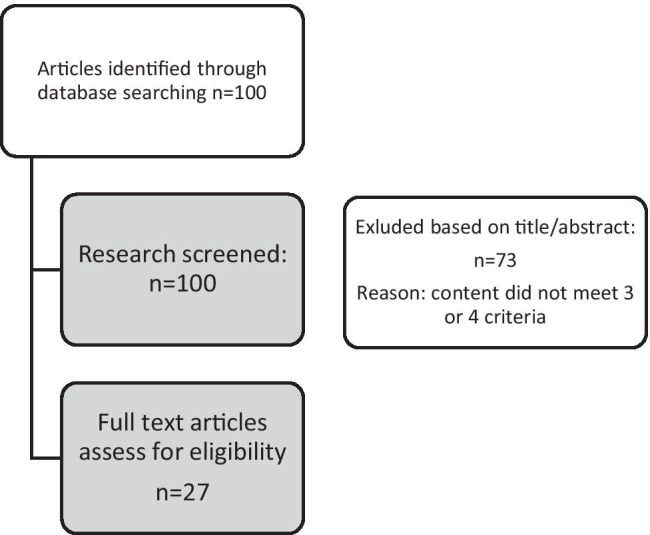
Selection procedure Search Part 1

The number of unique papers selected from the databases was 100 (see Appendix 1 for a table comprising all studies). A content evaluation of the abstracts of the selected papers was performed (see Fig. 1 for the selection process).

Cohen's *κ* was determined to assess level of agreement between the two reviewers. Specifically, the 100 papers and abstracts were assessed based on the three criteria outlined above (i.e., whether they concerned experience or interaction with nature, used digital representations of nature, and centred on a well-being-related outcome measure). Initially, the agreement between the two reviewers was moderate on the topic of nature interaction (*κ* = .46), perfect on the topic of digital nature (*κ* = 1), and substantial on the topic of well-being (*κ* = .67). After discussion between the reviewers, a substantial agreement on the topic of nature interaction (*κ* = .78) and an excellent agreement on the topic of well-being (*κ* = .86) was achieved. For the remaining 13 articles for which no agreement was reached, a third reviewer was involved to review these articles. Finally, agreement was achieved between the three reviewers, while the Cohen’s kappa remained stable (nature interaction (*κ* = .78), digital nature (*κ* = 1), well-being (*κ* = .86)).

### Study Characteristics

Only articles that covered at least three of the topics, according to research aim 1, were included in the in-depth analysis. Table 2 presents an overview of the 27 articles included and their main findings.

**Table 2:** Articles identified in Search Part 1

Reference	Title	Main findings
Astell-Burt et al. (2013)	Mental health benefits of neighbourhood green space are stronger among physically active adults in middle-to-older age: Evidence from 260,061 Australians	For adults in middle-to-older age, green spaces were identified as important for promoting physical activity, but the mental health benefits of greener environments appeared contingent upon active lifestyles.
Bell et al. (2015)	Seeking everyday wellbeing: The coast as a therapeutic landscape	Participants expressed strong and often enduring connections to the local coastline, with different coastal stretches perceived to serve varied therapeutic needs and interests, at multiple scales and intensities.
Bos et al. (2016)	A Primrose Path? Moderating Effects of Age and Gender in the Association between Green Space and Mental Health	Green space was associated with better mental health, but only in specific age and gender groups, and only in a 3-km, not a 1-km buffer. Moderating effects of age and gender were found and could explain whether or not people have the opportunity to make use of their green living environment.
Bruun-Pedersen et al. (2015a)	Simulating nature for elderly users—a design approach for recreational virtual environments	A set of guidelines with design considerations was presented that could be considered when designing recreational virtual environments. The guidelines combined considerations from tourism, urban and landscape design, psychology and virtual environment navigation guidelines.
Costello et al. (2019)	“A lot better than medicine”—Self-organized ocean swimming groups as facilitators for healthy ageing	This study explored the ways marine life, personal experiences and social connectedness influence use of public blue space. Findings highlighted that group membership promoted use of blue space, thereby increasing participants’ health and well-being, and supporting development of self-efficacy and resilience.
Dempsey et al. (2018a)	Coastal blue space and depression in older adults	This study showed that coastal blue space reduced depression amongst older adults via visual exposure rather than through physical proximity.
Egorov et al. (2017)	Vegetated land cover near residence is associated with reduced allostatic load and improved biomarkers of neuroendocrine, metabolic and immune functions	This study demonstrated beneficial effects of residential vegetated land cover on allostatic load and individual biomarkers. These findings are consistent with previously observed health benefits of exposure to urban vegetation and urban green spaces, including reduced levels of chronic stress, improved mental health, reduced risk of type 2 diabetes, cardiovascular disease, and premature mortality.
Grigsby-Toussaint et al. (2015)	Sleep insufficiency and the natural environment: Results from the US Behavioral Risk Factor Surveillance System survey	This study showed that access to the natural environment attenuated the likelihood of reporting insufficient sleep, particularly amongst men.
Guite et al. (2006)	The impact of the physical and urban environment on mental well-being	An association between the physical environment and mental well-being was shown in this study. The most important factors that operated independently were neighbour noise, sense of over-crowding in the home, and escape facilities such as green spaces and community facilities, and fear of crime.
Helbich (2019)	Dynamic urban environmental exposures on Depression and Suicide (NEEDS) in the Netherlands: a protocol for a cross-sectional smartphone tracking study and a longitudinal population register study	This study aimed to investigate whether and, if so, to what extent environments along people’s daily mobility and over their residential histories correlate with depression and suicide risk.
Helbich et al. (2018)	Natural environments and suicide mortality in the Netherlands: a cross-sectional, ecological study	Findings of this study showed that exposure to natural environments, particularly to greenery, might have a role in reducing suicide mortality.
Hunter et al. (2019)	Environmental, health, wellbeing, and social and equity effects of urban green space interventions: a meta-narrative evidence synthesis	This study provided supportive evidence regarding the use of certain urban green space (UGS) interventions for health, social and environmental benefits.
Kondo et al. (2020)	Momentary mood response to natural outdoor environments in four European cities	The findings of this study showed evidence of psychological and mental health benefits of exposure to natural outdoor environments, especially amongst urban populations.
Lee and Lee (2019)	Do sociodemographic factors and urban green space affect mental health outcomes among the urban elderly population?	This study showed that the prevalence of mental health issues generally decreased in relation to the ratio of green space in an area. The authors suggest that the ratio of urban green space within a community is an important component in improving mental health outcomes for elderly urban residents.
Mukherjee et al. (2017a)	Park availability and major depression in individuals with chronic conditions: Is there an association in urban India?	The authors concluded in this study that availability of large parks in the immediate neighbourhood positively impacted mental well-being of individuals with pre-existing chronic conditions. The authors stressed that to promote health through smart urban design, urban green spaces should be included.
Nakau et al. (2013)	Spiritual care of cancer patients by integrated medicine in urban green space: a pilot study	The study showed that a spiritual program offered in an urban green space improved quality of life and reduced cancer- associated fatigue in cancer patients.
Noordzij et al. (2020)	Effect of changes in green spaces on mental health in older adults: a fixed effects analysis	In this study observed cross-sectional correlations between the accessibility of green space in the residential environment and mental health were found, but no evidence was found for an association between changes in green spaces and changes in mental health.
Nutsford et al. (2013)	An ecological study investigating the association between access to urban green space and mental health	This study showed that decreased distance to useable green space and increased proportion of green space within the larger neighbourhood were associated with decreased anxiety and reduced prevalence of mood disorders in an urban environment.
Patel et al. (2019)	Green space and mental health symptoms in a cardiac rehabilitation population	The results of this study suggested that increased accessible green space near the home may improve depression and promote recovery in a cardiac rehabilitation population.
Pun et al. (2018)	Association of neighborhood greenness with self-perceived stress, depression and anxiety symptoms in older US adults	A direct association of greenness with lower perceived stress amongst older adults, and an indirect association mediated through physical activity and respiratory disease history was found in this study.
Triguero-Mas et al. (2017)	Natural outdoor environments and mental health: Stress as a possible mechanism	This study indicated that contact with natural outdoor environments benefits mental health and having contact with these environments can facilitate stress reduction.
van den Bosch et al. (2015)	Moving to serene nature may prevent poor mental health—results from a Swedish longitudinal cohort study	In this study there was no significant correlation between pre- and post move to “serene” nature and change in mental health. However, the specific quality “serene nature” significantly decreased risk for mental health issues amongst women.
Vogt et al. (2015)	Neighborhood and healthy aging in a German city: distances to green space and senior service centers and their associations with physical constitution, disability, and health-related quality of life	In this study the expected association between distance to the nearest green space and healthy aging was not found. The authors argued that this finding might relate to the high proportion of greenness in this study’s location.
White et al. (2018)	A prescription for “nature”—the potential of using virtual nature in therapeutics	This study showed that while contact with real-world nature is preferred, virtual reality representations of nature can be an alternative in cases when in vivo contact with nature is not possible.
Zhang et al. (2015)	Green space attachment and health: a comparative study in two urban neighbourhoods	In this paper greater attachment to local green space and better self-reported mental health were found when participants had higher availability of accessible and usable green spaces in their neighbourhood.
Zhang et al. (2019)	Objectively measured neighbourhood attributes as correlates and moderators of quality of life in older adults with different living arrangements: the ALECS Cross-sectional study	This study showed that older adults living alone in neighbourhoods with poor access to destinations and few activities in parks demonstrated lower environmental and/or social quality of life than their counterparts.
Zijlema et al. (2017)	The relationship between natural outdoor environments and cognitive functioning and its mediators	This study indicated that proximity to nature may benefit cognitive functioning, but the authors could not establish which mechanisms may explain this relationship.

It is important to note that although 27 studies were selected which met at least three of four criteria, only two studies met all four of the criteria and describe research on virtual reality representations of nature to improve well-being for older adults.

To sum up, the aim of this review was to identify current insights in studies on benefits of virtual reality representations of nature on well-being for elderly. Since only 2 of the 27 selected papers actually met all four criteria, we feel safe to conclude that there is a lack of integration of insights across the four different topics. We decided to run a second search with a stronger focus on connectedness (rather than well-being) to identify relevant studies on social aspects of well-being missed in the first round.

#### Search Part 2

The search key words (arranged per topic) are presented in Table 3.

**Table 3:** Search key words per topic (Search Part 2)

Elderly	Technology	Connectedness	Nature
“Elderly”	Technolog*	“Social connectedness”	“Nature experience”
“Aged”	System	“Connectedness”	“Nature exposure”
“Older”	“Virtual reality”	“Loneliness”	“Restorative nature”
“Elder”	“VR”	“Social isolation”	“Nature environment”
“Geriatric”	“Augmented reality”	“Mental health”	“Green space”
“Elderly people”	“Ambient technology”	Psycholog*	“Blue space”
“Old people”	“Pervasive technology”		
“Senior”			

#### Results Part 2

**Fig. 2:**
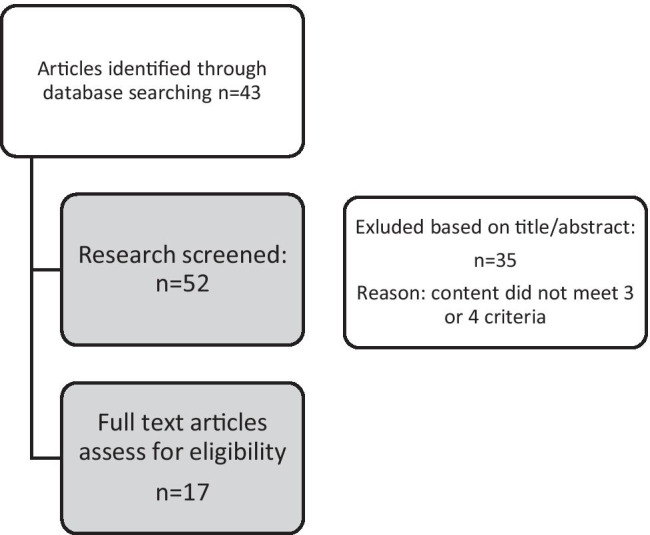
Selection procedure Search Part 2

We performed a content evaluation on the abstracts of the selected papers of the search combining all topics (Elderly & Nature & Connectedness & Technology). Figure 2 shows the selection process.

After checking for duplicates, 52 articles remained. A table representing all selected articles (and topics covered), that were not selected in part 1, is presented in Appendix 2.

### Study Characteristics

Similar to part 1, in search part 2 only studies that met at least three of the criteria were included in the in-depth analysis. Only one new study was identified (see Table 4); 16 studies were identified that were already discussed in Table 2 (Astell-Burt et al., 2013; Bos et al., 2016; Bruun-Pedersen et al., 2015a; Dempsey et al., 2018b; Egorov et al., 2017; Grigsby-Toussaint et al., 2015; Helbich et al., 2018; Lee & Lee, 2019; Mukherjee et al., 2017a; Nakau et al., 2013; Nutsford et al., 2013; Pun et al., 2018; Van den Berg et al., 2016; White et al., 2018; Y. Zhang et al., 2015; Zijlema et al., 2017).

**Table 4:** Articles identified in Search Part 2

Reference	Title	Main findings
Akpinar et al. (2016)	Does green space matter? Exploring relationships between green space type and health indicators	This study suggested that types of green space should be considered individually rather than aggregated as “simply green.” Additionally, “size” of forest in urban areas seemed an important factor impacting the relationship between green space and mental health.

Similar to search part 1, only two articles describe research on virtual reality representations of nature to improve well-being for older adults; the same articles identified in search part 1 (i.e., White et al. [2018] and Bruun-Pedersen et al. [2015a]).

To conclude, the aim of this search was to identify current insights in studies on benefits of virtual reality representations of nature on well-being of elderly. In the final search, we decided to redefine our search and exclude the search key words of Connectedness with the aim to identify more technology-related articles and repeat the database search.

#### Search Part 3

For this search, we used the search key words and synonyms for Technology, Nature, and Elderly. Other than that, the search strategy was equal to the previous searches.

#### Results Part 3

After checking for duplicates, the number of unique articles selected from the databases was 143. The table of all hits that were not included in part 1 or part 2 is presented in Appendix 3.

### Study Characteristics

Only articles that met all three criteria were included in the in-depth analysis. Two studies were identified, of which 1 (White et al., 2018) was already described in Table 2. Table 5 presents the remaining included study and its main findings.

**Table 5:** Articles identified in search part 3

Reference	Title	Main findings
Battisto et al. (2018)	Technological supports to increase nature contact for older adults	This study stressed the opportunities to utilize technologies for connecting older adults to nature and described challenges related to the creation of immersive, high fidelity, realistic nature settings which could function as a substitute for contact with real nature.

**Table 6:** Table search part 1

	Nature interaction	Technology	Well-being	Older adults (not excluded)
Astell-Burt et al. (2013)	x		x	x
Astell-Burt et al. (2016)	x		x	x
Bell et al. (2015)	x	GPS	x	x
Bentsen et al. (2009)	x		x	
Bentsen et al. (2010)	x			
Bernatzky (1975)	x		x	
Bodin et al. (2015)		GPS	x	x
Boeyen et al. (2017)	x			
Bos et al. (2016)	x		x	x
Botticello et al. (2015)		GPS		x
Bruun-Pedersen et al. (2015a)	x	x	x	x
Büssing et al. (2005)			x	x
Cartwright et al. (2018)	x		x	
Claessens et al. (2014)	x	GPS		
Costello et al. (2019)	x		x	x
Coutts et al. (2013)	x			x
Dempsey et al. (2018a)	x		x	x
Dzhambov et al. (2018)	x	GPS		
Eckenwiler (2018)				
Egorov et al. (2017)	x	GPS	x	x
Engemann et al. (2019)	x	GPS	x	
Ferrara et al. (2019)	x	GPS		x
Foster et al. (2009)	x	GPS		x
de Gelder et al. (2017)				
Generaal et al. (2019)		GPS	x	
Germenis (2014)				
Goyder et al. (2014)		GPS		x
Grazuleviciene et al. (2016)	x			x
Grigsby-Toussaint et al. (2015)	x		x	x
Guite et al. (2006)	x		x	x
Helbich (2019)	x	GPS	x	x
Helbich et al. (2018)	x	GPS	x	x
Helbich et al. (2016)	x	GPS		
Huang et al. (2020)	x			x
Hunter et al. (2019)	x		x	x
Huynh et al. (2013)	x	GPS	x	
Jansen et al. (2017)	x	GPS		x
Kessel et al. (2009)	x	GPS		
Kondo et al. (2017)	x	GPS		
Kondo et al. (2020)	x	GPS	x	x
Kumagai et al. (2015)				
Lanki et al. (2017)	x			
Lee and Lee (2019)	x		x	x
Li and Ghosh (2018)	x	Google Street View		x
Liang et al. (2017)	x	GPS		
Liao et al. (2019)	x			
Liao et al. (2019a, b)	x			
Liddicoat et al. (2020)				
Lin et al. (2018)	x	GPS		
Logan et al. (2015)	x		x	
Lyu et al. (2018)	x		x	
Ma et al. (2018)	x	GPS		x
Magalhães et al. (2017)	x			
Mäki-Opas et al. (2016)	x	GPS		x
Manferdelli et al. (2019)				
McGrath et al. (2015)	x	GPS		
Miralles-Guasch et al. (2019)	x	GPS		x
Mukherjee et al. (2017b)	x	GPS	x	x
Müller et al. (2018)	x	GPS		x
Mygind et al. (2018)	x		x	
Nakau et al. (2013)	x		x	x
Nichani et al. (2017)	x	GPS	x	
Noordzij et al. (2020)	x		x	x
Nordbø et al. (2019)	x	GPS	x	
Nutsford et al. (2013)	x		x	x
Ord et al. (2017)	x			x
Paquet et al. (2013)	x			x
Park (2017)	x	GPS		
Patel et al. (2019)	x	GPS	x	x
Pereira et al. (2019)	x			
Puhakka et al. (2018)	x	GPS		
Pun et al. (2018)	x		x	x
Rahman and Zhang (2018)	x	Google Earth		x
Reid et al. (2009)				x
Ribeiro et al. (2019)	x		x	
Rook (2013)	x		x	
Rook et al. (2014)	x		x	
Servadio et al. (2019)				
Stewart et al. (2018a)	x	GPS		x
Storgaard et al. (2013)	x	GPS		x
Su et al. (2019)	x	GPS	x	
Sugiyama et al. (2016)	x		x	
Sun et al. (2017)		GPS		
Tan et al. (2007)				x
Triguero-Mas et al. (2017)	x		x	x
van den Bosch et al. (2015)	x	GPS	x	x
Vienneau et al. (2017)	x			
Vogt et al. (2015)	x	GPS	x	x
Wang et al. (2017a, b)	x			x
Wang et al. (2019)	x	GPS		x
Wang et al. (2019a, b)				x
White et al. (2018)	x	x	x	x
Younan et al. (2018)				
Zandieh et al. (2019)	x	GPS		x
Zhang et al. (2015)	x		x	x
Zhang et al. (2019)	x	GPS	x	x
Zijlema et al. (2017)	x		x	x

**Table 7:** Table search part 2 (excluding articles selected in part 1)

	Nature	Technology	Connectedness	Older adults (not excluded)
Akpinar et al. (2016)	x		x	x
Ashbullby et al. (2013)	x		x	
Debele (2014)				
Moffat et al. (2009)	x			
Villeneuve et al. (2012)	x			
Younan et al. (2016)	x		x

**Table 8:** Table search part 3 (excluding articles selected in part 1 or 2)

	Nature	Technology	Older adults (not excluded)
Almeter et al. (2018)	x	GPS	
Astell-Burt et al. (2016)	x	GPS	x
Barbosa et al. (2007)	x	GPS	x
Battisto et al. (2018)	x	x	x
Benmarhnia et al. (2017)			x
Bunney et al. (2016)	x	GPS	
Burgoine et al. (2015)	x	GPS	
Cassarino et al. (2019)	x	x	
Chien et al. (2019)		GPS	x
Cochrane et al. (2009)	x	GPS	x
Coutts et al. (2010)	x	GPS	x
Cui et al. (2013)	x		
Cumo et al. (2017)	x		x
Dadvand et al. (2017)	x		
Douglas et al. (2018)	x	GPS	x
Forsyth and Crewe (2010)	x		
Gao et al. (2012)	x		
Germann-Chiari and Seeland (2004)	x	GPS	x
Gose et al. (2013)		GPS	
Graça et al. (2018)	x		x
Green et al. (2016)	x		
Haggag (2010)	x		
Hermida et al. (2017)	x	GPS	x
Hillsdon et al. (2006)	x	GPS	x
Hoffimann et al. (2017)	x	GPS	
Huang et al. (2018)	x		x
Hui et al. (2017)	x		
Jamaludin et al. (2014)	x		
Janssen and Rosu (2015)	x	GPS	
Jim and Chen (2007)	x		
Jim and Shan (2013)	x		x
Jones (2018)	x		
King et al. (2015)	x		x
King et al. (2012)	x	GPS	x
Kruuse Afverchou (2005)			
Lachowycz et al. (2012)	x	GPS	
Li and Ghosh (2018)	x	Google Street View	x
Li et al. (2008)			
Lin et al. (2018)	x	GPS	
Liu et al. (2017)	x		x
Marquet et al. (2019)	x	GPS	
Michimi and Wimberly (2012)	x	GPS	x
Mihrshahi et al. (2018)			
Møller et al. (2019)	x	GPS	
Morris et al. (2006)			
Ngom et al. (2016)	x	GPS	x
Occhiuto (2018)	x		
Othman et al. (2015)	x	x	
Panyadee et al. (2016)	x		x
Potestio et al. (2009)	x	GPS	
Pourzargar (2016)	x		
Prince et al. (2011)	x		x
Rahman et al. (2019)	x	GPS	
Rahman and Zhang (2018)	x	GPS	x
Raymond et al. (2016)	x	GPS	x
Ribeiro et al. (2015)	x	GPS	x
Richardson et al. (2017)	x	GPS	x
Rudnev (2012)	x		
Saghafi and Ahmadpour (2017)	x		x
Sanchez et al. (2010)	x		x
Shackleton et al. (2015)	x		x
Shourbela et al. (2016)			
Shrestha et al. (2018)	x		
Son et al. (2019)	x		x
Stewart et al. (2018b)	x	GPS	x
Sugiyama et al. (2014)	x	GPS	x
Sun et al. (2017)	x	GPS	x
Tan et al. (2007)	x		x
Tian and Jim (2012)	x		
Tian et al. (2014)	x		
Tikka et al. (2000)	x		
Uitto et al. (2006)	x		
Veitch et al. (2015)	x	GPS	x
Wang et al. (2017a, b)	x		x
Wang et al. (2016)	x	GPS	x
Wang et al. (2019a, b)	x	GPS	x
Wang et al. (2017)			
Wang and Liu (2017)	x		
Xie et al. (2018)	x		
Xu and Gao (2017)	x		x
Xu et al. (2019)	x		
Zacharias et al. (2015)			
Zhu and Jia (2012)	x		

Only three articles were identified that met all search criteria. Next, we will discuss these articles in more detail to generate understanding of the current knowledge base within the field of virtual reality research and digital nature representation.

In the article of Bruun-Pedersen et al. (2015b), a design approach for recreational virtual nature for elderly is proposed, with the possibility of implementation in rehabilitation health settings. This article is a follow-up on a pilot study in which nursing home residents were exposed to an augmented alternative for their daily biking exercise to improve physical well-being (Bruun-Pedersen et al., 2014). In this study, the authors proposed a set of guidelines with design considerations such as navigation guidelines and guidelines for content types of potential nature landmarks which might be used in recreational virtual environments. The authors conclude that the guidelines are based on literature and need further testing in real life settings.

The second article by White et al. (2018) is a review article on the possible uses of virtual nature in therapeutics to improve quality of life. The authors argue that when real interaction with nature is not possible or feasible, for example, for elderly with mobility issues, virtual nature could be considered as an alternative. The authors mention that there are several implementation possibilities of virtual nature or virtual reality in general in health environments. White et al. further recommend to also keep in mind the risks, benefits, and cost efficiency of these implementations but do not further describe them in much detail in their article.

The last study identified by our review that met all criteria is the study by Battisto et al. (2018). In this article, the authors discuss technological possibilities to increase nature interaction for older adults. They argue that technology could be used to make therapeutic landscapes accessible for older adults to promote health and to improve quality of life (Battisto et al., 2018). Subsequently, several examples of implementations are discussed, such as simulations, virtual nature environments, and interactive displays. According to Battisto et al. (2018), more research is needed in the field, and advanced technological solutions should be developed, especially for the design of convincing and realistic settings that provide the user with a feeling of actually being present in the virtual environment.

In conclusion, the three studies identified generate preliminary evidence for the effectiveness of virtual (nature) environments as a means to improve well-being amongst older adults. These studies provide starting points for the design and (technological) development of such environments. However, as of yet, there is no evidence-based design approach that generates understanding of how specific characteristics of virtual nature environments impact social well-being measures in particular, and of the underlying psychological processes involved.

## Discussion

In the present paper, a rapid literature review consisting of three parts was reported with the aim to identify articles that focus on the effects of nature interaction on (social) well-being for older adults, and specifically, articles focussing on virtual representations of nature for older adults as a means to improve social well-being. In total, 29 unique articles were identified across the three searches that met at least three of the four criteria (aim 1). Of these 29 articles, only three articles were identified using virtual representations of nature for elderly focussing on promoting general health (Battisto et al., 2018), recreation, and rehabilitation (Bruun-Pedersen et al., 2015b) and quality of life (White et al., 2018). None of these articles, however, specifically aimed at improving social well-being of elderly users.

As such, the searches reported on in the present undertaking clearly point at a blind spot in contemporary research. Whereas there is a considerable body of research when zooming in on the research topics in isolation, there is very little cross-disciplinary research combining these topics by connecting insights from the social sciences with technology research and development. This connection is essential for successful implementation of virtual representations of nature in the lives of older adults.

Articles identified in this review mostly focus on the effects of (nearby) green space and mental health, such as reducing stress or improving quality of life. This body of research underscores the importance of (amongst others) available, nearby or urban green space for the mental health of the (ageing) population. These studies, however, do not contribute to solutions or innovations that make nature accessible for those with limited access to nature. Although many studies were identified using GPS or GIS data for data collection, only an extremely small number of studies using other types of technologies were identified. When considering the many ways in which digital nature could be presented to older adults using diverse technologies (such as virtual or augmented reality, smart screens, interactive walls, smart projections and so on), research exploring and testing effects (also taking into account frequency and duration of exposure) is highly called for.

Clearly, future research is warranted to unravel which digital types of nature could improve well-being for older adults, and to what extent such interventions can remedy social well-being (including loneliness and feelings of connectedness) in particular. In terms of urgency, bringing nature inside would be especially beneficial to older adults with mobility issues and to those living in urbanized regions where nature is scarce (Battisto et al., 2018; Browning et al., 2020; White et al., 2018).

Finally, the present review (including the three studies identified in the final search iteration) did not yield insights as to what specific virtual representations of nature characteristics are associated with improvements in (social) well-being. We aimed to identify studies reporting on preferences of older adults not only in real life nature (cf. Wen et al., 2018), but especially within virtual nature environments. According to a review article (Depledge et al., 2011), landscape features tested most frequently within virtual environments are concrete elements such as trees, people, and water. However, their effects on social well-being in particular were not tested. Additionally, these studies do not aim at identifying how more abstract visual-spatial characteristics such as spatial configuration, spaciousness, and perceived enclosure in digital nature environments impact outcome measures. Although specific visual-spatial features in augmented nature scenes like spaciousness have been shown to influence social aspirations within a student population (van Houwelingen-Snippe et al., 2020a, b), research is needed to identify whether such characteristics can also enhance (social) well-being and related measures amongst older adults.

Specifically related to the present pandemic (COVID-19), social and mental well-being problems are predicted to aggravate in the upcoming period (Simon et al., 2020). These specific times bring many challenges with them for everyone, but especially so for older adults who are generally more vulnerable and for whom going outside might be even more of a challenge. When also considering the many restrictions worldwide, the importance of virtual representations of nature for older adults cannot be overstated.

## Limitations

The number of studies matching all criteria was extremely limited. Table 2 indicates that although the total number of papers found with the isolated topics was substantial, clearly this was not the case for papers combining multiple topics. Hence, our findings call for multidisciplinary research approaches integrating findings from the domains of gerontology, nature research, and human media interaction research. Considering the limited number of papers, we did not include additional criteria (e.g., type of study, strength of evidence) to control for quality and relevance of the selected papers.

Arguably, our search key words were rather broad (e.g., the search key words concerning Elderly), which may have resulted in a failure to identify papers targeted at very specific patient groups or papers targeting age-related health problems including dementia or Parkinson’s disease. For these patient groups, however, digital nature is often used as a means of recovery from fatigue or for recreational purposes (e.g., visiting a tropical island as a welcome distraction from daily concerns), rather than as a means for improving social well-being.

## Conclusions

This rapid review points at a lack of studies combining insights of geriatric studies, nature studies, and human-system interaction studies. Considering the diverse benefits of contact with nature to an ageing population and the many possibilities smart technologies provide for bringing nature inside, this review shows that opportunities for challenging, boundary-spanning research approaches to one of the most pressing societal challenges of our times are many.

## Appendix 1

## Appendix 2

## Appendix 3
